# Association between quarantine and sleep disturbance in Hong Kong adults: The mediating role of COVID-19 mental impact and distress

**DOI:** 10.3389/fpsyt.2023.1127070

**Published:** 2023-02-28

**Authors:** Ted C. T. Fong, Kay Chang, Rainbow T. H. Ho

**Affiliations:** ^1^Centre on Behavioral Health, Faculty of Social Sciences, The University of Hong Kong, Pokfulam, Hong Kong SAR, China; ^2^Department of Psychology, Faculty of Social Sciences, University of Macao, Macao, Macao SAR, China; ^3^Department of Social Work and Social Administration, Faculty of Social Sciences, The University of Hong Kong, Pokfulam, Hong Kong SAR, China

**Keywords:** Chinese, COVID-19, pandemic, psychological distress, quarantine, sleep problems

## Abstract

**Background:**

COVID-19 quarantine has been associated with increased sleep problems and prolonged psychological responses to the pandemic could mediate this relationship. The present study attempted to examine the mediating role of COVID-19 mental impact and distress between quarantine and sleep disturbance.

**Methods:**

The present study recruited 438 adults (109 with quarantine experience) in Hong Kong *via* an online survey between August and October 2021. The respondents completed a self-report questionnaire on quarantine, Mental Impact and Distress Scale: COVID-19 (MIDc), and Pittsburgh Sleep Quality Index (PSQI). The MIDc was treated as a latent mediator and continuous PSQI factor and poor sleep quality (PSQI score > 5) were the study outcomes. We evaluated the direct and indirect effects of quarantine on sleep disturbance *via* MIDc using structural equation modeling. Analyses were adjusted for gender, age, education level, knowing confirmed COVID-19 cases, COVID-19 frontline work, and primary income source of the family.

**Results:**

More than half (62.8%) of the sample reported poor sleep quality. Quarantine was associated with significantly higher levels of MIDc and sleep disturbance (Cohen *d* = 0.23 – 0.43, *p* < 0.05). In the structural equation model, the MIDc mediated the relationship between quarantine and sleep disturbance (*αβ* = 0.152, 95% CI = 0.071 to 0.235). Quarantine significantly increased the proportion of poor sleep quality by 10.7% (95% CI = 0.050 to 0.171) indirectly *via* MIDc.

**Conclusions:**

The results provide empirical support to the mediating role of the MIDc as psychological responses in the relationship between quarantine and sleep disturbance.

## Introduction

Coronavirus disease 2019 (COVID-19) is a global pandemic that caused 6.6 million deaths since early 2020. The COVID-19 pandemic caused various mental health problems such as anxiety, depression, social stigma, and mental stress across different populations in general (1–3). Given its contagious nature and fatality (4), various policies such as border controls, contact tracing, social distancing, and restriction of community activities (5) were used to curb the spread rate of coronavirus worldwide. Mandatory quarantine is defined as compulsory separation and restriction of movement of people who have potentially been exposed to the coronavirus. Though quarantine measures reduced the risk of the exposed individuals infecting others, individuals under quarantine often experienced a loss of life routine and social interaction, leading to possible social isolation. The quarantine experience has been associated with self-stigma, loneliness, and psychological distress during the pandemic (6, 7). A number of stressors associated with quarantine such as longer quarantine duration, boredom, fear of infection, and financial loss could aggravate the psychological impact (6) and have been linked with depression and sleep disorders (8).

Sleep is an essential physiological indicator for physical and mental health. Insufficient sleep and poor sleep quality have been shown to predict various medical morbidities (9). Recent meta-analyses (10, 11) estimated the global prevalence rate of sleep problems to be 18–32.3% among the general population and 57–74.8% among COVID-19 cases. A recent meta-analytic study (12) showed that female sex is an important predictor of sleep disturbances during the context of COVID-19 pandemic. Persons under quarantine are prone to an unprecedented stressful situation of home confinement. Previous studies have revealed altered sleep patterns among quarantined individuals under a prolonged stay-at-home situation during the outbreak (13, 14). Hong Kong is a fast-paced metropolitan city characterized by long working hours and stressful workplace culture. Sleep problems have been a prevalent phenomenon with a previous survey (15) reporting a prevalence rate of 60.4% for poor sleep quality in primary care patients aged over 40 years in Hong Kong. Recent studies (16, 17) have found elevated rates of poor sleep quality among samples of Hong Kong adults between 2019 and 2020.

The majority of the literature found a negative impact of COVID-19 quarantine on sleep and psychological distress (10, 18–20). The prevalence of sleep problems during quarantine was estimated to range from 29 to 76% among various adults and adolescents samples in European countries (Italy and France), Middle Eastern countries (Saudi Arabia, Israel, and Jordan), and South American countries (Columbia and Brazil) (18, 21–26). In 2021, Hong Kong has one of the longest and strictest quarantine measures with a 21-day quarantine period at hotels or dedicated quarantine facilities. A recent study in Hong Kong (27) found negative impacts of quarantine on mental health and sleep outcomes among 248 participants under mandatory hotel quarantine. However, no existing studies have taken specific account of the quarantine effects on potential disruptions in sleep patterns. It is of practical importance to better understand the linkage between quarantine and sleep disturbance.

Individuals' psychological responses to COVID-19 have been assessed by existing standardized scales such as the Patient Health Questionnaire and Generalized Anxiety Disorder Scale in recent studies (28, 29). Though their use facilitated comparison of results across different studies, these scales could not assess pandemic-specific impacts such as fear of virus infection, shortage of necessities and food, and financial insecurity due to mandatory quarantine. Uncertainty regarding possible COVID-19 infection and fear of direct contact with confirmed COVID-19 cases have been linked with higher risks of sleep disturbance (30). The Mental Impact and Distress Scale: COVID-19 (MIDc) has recently been developed to examine the perceived situational impact and psychological responses associated with the COVID-19 pandemic (31). The MIDc assesses two types of psychological responses, namely, anticipation and modulation, as a result of COVID-19. Anticipation refers to the mental engagement with one's active coping in response to the perceived threat of the pandemic, while modulation refers to the activation of mental insulation processes to maintain a sense of normalcy as a passive response to the immediate or heightened threat.

Though the MIDc has demonstrated satisfactory construct validity and convergent validity with anxiety, depression, and wellbeing in the Brazilian context, less is relatively known on the relationship between the MIDc and sleep disturbance. Empirical research is required to elucidate the potential mechanisms in which the quarantine experience leads to sleep disturbance and MIDc could play a mediating role in this relationship. In light of the research gaps, the present study had three research objectives. First, we aimed to calculate the prevalence of poor sleep quality among Hong Kong adults during the COVID-19 pandemic. Second, we aimed to evaluate the effect of quarantine on mental impact and distress and sleep disturbance. Third, we aimed to examine the potential mediating role of the MIDc in the relationship between quarantine and sleep disturbance. There were two main study hypotheses. First, we hypothesized that the quarantine experience would be significantly and positively associated with MIDc and sleep disturbance. Second, we hypothesized that MIDc would substantially mediate the association between quarantine and sleep disturbance.

## Methods

### Study design and procedures

The present study recruited 438 community adults in Hong Kong *via* an online survey under convenience sampling between August and October 2021. This period was situated before the fifth COVID-19 driven by the Omicron variant and only 362 new COVID-19 cases were reported in Hong Kong during the three-month interval. Despite the low number of infected cases, the government repeatedly locked down residential buildings with multiple COVID-19 cases to conduct mass testing. Individuals who were close contacts of confirmed or suspected cases were forced to undergo mandatory quarantine. Eligibility criteria included at least 18 years of age, residing in Hong Kong, and ability to understand written Chinese.

The research team used mass emails and websites of local universities as promotional means to facilitate recruitment in the community. In total, three rounds of mass emails were sent to invite potential participants from the local universities and community to fill in the online survey. The participants were presented with the survey nature before providing their informed consent. Participation was strictly voluntary and the participants could withdraw from the survey without negative consequences. Contact information of emotional support services was provided in the end of the survey for the participants to seek help in case of discomfort. Ethical approval was obtained from the human research ethics committee of the authors' university in July 2021 (Reference numbers: EA210291). The participants completed a self-report questionnaire on demographic characteristics, quarantine experience, COVID-19 mental impact and distress, and sleep disturbance.

Power analysis was conducted using Monte Carlo simulation techniques to determine the sample size requirements for mediation analysis (32). A structural equation model was specified with three observed indicators each measuring the predictor, mediator, and outcome variables as latent factors (λ = 0.8). Standardized path coefficients of 0.14 and 0.39 corresponded to small and moderate (2 and 13% of the variance) effect sizes (33). Assuming a direct effect with small to moderate magnitudes, the present sample size (*N* = 438) showed excellent statistical power (100%) for detecting indirect effects with moderate magnitudes (*a* = *b* = 0.39). For indirect effects with small to moderate magnitudes (*a* = 0.39, *b* = 0.14 or *a* = 0.14, *b* = 0.39), the statistical power dropped to 61.6–66.8%.

### Measures

The present study assessed the quarantine experience of the respondents *via* a single item on whether they had previously gone through any government mandatory quarantine due to COVID-19. The questionnaire inquired about demographic characteristics such as age, gender, and education level, and pandemic-related characteristics on whether (1) they personally knew anyone who had been diagnosed with COVID-19; (2) they took part in COVID-19 frontline work such as health care and essential services; (3) they are the source of primary income of their family.

Sleep disturbance was measured by the Pittsburgh Sleep Quality Index (PSQI) (34). The 19-item PSQI evaluated the degree of sleep disturbance perceived by the participants through seven components, namely, subjective sleep quality, sleep latency (time between lying down on bed and falling asleep), sleep duration, habitual sleep efficiency, sleep disturbance, use of sleep medication, and daytime dysfunction over the past month. The seven components were rated on a 4-point Likert scale from 0 to 3 and added together to produce the global sleep disturbance score (theoretical range = 0–21). A global PSQI score greater than 5 denoted poor sleep quality. The Chinese version of the PSQI has been validated with good psychometric properties in a previous study in Hong Kong (35). The PSQI showed satisfactory reliability (*α* = 0.76) in the present sample.

The Mental Impact and Distress Scale: COVID-19 (MIDc) was a 3-factor trans-diagnostic assessment tool of mental impact and distress associated with COVID-19 (31). The three factors of the MIDc included situational impact, anticipation, and modulation. Situational impact was an 8-item subscale that evaluated respondents' perceived impact of COVID-19 on functioning in the health, work, financial, and interpersonal domains. Anticipation (10 items) and modulation (9 items) were two other factors that measured psychological responses of the respondents as a result of COVID-19. Sample items of anticipation included “I am preoccupied with thoughts of how to stay out of danger” and “I think about the situation when I don't mean to”, and sample items of modulation included “I have trouble concentrating” and “I feel exhausted”. All MIDc items were scored on a 5-point Likert scale from 1 (“not me at all”) to 5 (“totally me”). The total MIDc score and subscale scores of situational impact, anticipation, and modulation had a theoretical score from 27 to 135, 8 to 40, 10 to 50, and 9 to 45, respectively. The Chinese version of the MIDc has been validated in a recent study in Hong Kong (36). The three subscales showed good reliability (*α* = 0.83–0.93) in the present sample.

### Data analysis

Chi-square tests and independent t-tests were conducted using SPSS 26.0 to compare the demographics and psychological profiles across quarantine experience. Statistical significance was set at 0.05 in the present study. Effect size of the *t*-tests was denoted by Cohen d, with cutoffs of 0.2, 0.5, and 0.8 denoting small, medium, and large effect sizes, respectively. Three of the seven PSQI components showed substantial floor effects (over 25% of the respondents endorsing the minimal level) and all PSQI components were treated as ordinal variables. Confirmatory factor analysis (CFA) was conducted using Mplus 8.4 (37) to examine the 3-factor structure of the MIDc and 1-factor structure of the PSQI under the robust weighted least square estimator. Model fit was evaluated based on the following cutoff criteria (38): comparative fit index (CFI) ≥ 0.95, root mean square error of approximation (RMSEA) ≤ 0.06, and standardized root mean square residuals (SRMR) ≤ 0.06. The data analyzed in this study are available in a Supplementary file.

In the structural equation model (SEM), quarantine experience was posited as the binary observed predictor. The total MIDc factor was posited as a latent mediator measured by the three subscales (situational impact, anticipation, and modulation). For the outcome variable, sleep disturbance was examined both as a continuous latent factor measured by the seven PSQI components and an observed binary item on poor sleep quality (global PSQI score > 5). The control variables of the SEM included demographic and other pandemic-related characteristics. Direct and indirect effects of quarantine on sleep disturbance *via* MIDc were evaluated in the SEM. R-square denoted the proportion of explained variance of the dependent variables. To accommodate the likely skewed distribution, the indirect effects were estimated using 10,000 bootstrap draws and regarded as statistically significant if the 95% confidence interval (CI) excluded zero. Sensitivity analyses were conducted by repeating the SEM analyses across gender (females and males) and age groups (18–39 years and ≥ 40 years). There were no missing data in the MIDc and PSQI items and four participants had missing data on the control variables. Missing data were handled using full information maximum likelihood under the missing-at-random assumption (39).

## Results

### Sample profile

The majority of the respondents were women (75.9%) and had tertiary education level (89.3%) with a mean age of 38.8 years (SD = 13.9). Three-tenths (29.7%) of the sample personally knew confirmed COVID-19 case and two-fifths (41.1%) were the primary income source of the family. One-fourth (24.9%) of the sample experienced compulsory government quarantine and one-twelfth (8.2%) took part in COVID-19 frontline work. Overall, the sample displayed moderate leveLs of situational impact, anticipation, and modulation (*M* = 17.3–18.4, SD = 5.75–7.84). The sample reported a mean of 7.21 (SD = 3.75) for the global PSQI score and five-eighths (62.8%) of the sample reported poor sleep quality. Both the PSQI and MIDc scores were positively skewed (skewness = 0.70–1.38). As Table 1 shows, there were no significant gender differences in the study variables except for age (*d* = 0.25, *p* < 0.05). Compared to the younger age group, the older age group showed significantly lower levels of mental impact and distress associated with COVID-19 (*d* = 0.19–0.33, *p* < 0.05).

**Table 1:** Demographics and psychological profiles of the sample across gender and age subgroups.

**Variables**	**Females**	**Males**		**Age 18-39**	**Age ≥40**	
	**(*N =* 331)**	**(*N =* 105)**		**(*N =* 237)**	**(*N =* 199)**	
**Categorical variable**	***N*** **(%)**	***N*** **(%)**	*χ^2^*	***N*** **(%)**	***N*** **(%)**	*χ^2^*
Education level			1.29			49.9^**^
Secondary	36 (10.9)	10 (9.5)		13 (5.5)	33 (16.6)	
Bachelor	143 (43.2)	52 (49.5)		142 (59.9)	54 (27.1)	
Master or above	152 (45.9)	43 (41.0)		82 (34.6)	112 (56.3)	
Know confirmed case	105 (31.7)	24 (22.9)	3.01	58 (24.5)	72 (36.2)	7.09^**^
Frontline work	28 (8.5)	8 (7.6)	0.07	15 (6.3)	21 (10.6)	2.55
Family's primary income	135 (40.8)	45 (42.9)	0.14	69 (29.1)	110 (55.3)	30.6^**^
PSQI diagnosis	207 (62.5)	67 (63.8)	0.06	150 (63.3)	123 (61.8)	0.1
**Continuous variable**	**Mean (SD)**	**Mean (SD)**	***d***	**Mean (SD)**	**Mean (SD)**	***d***
Age	39.7 (13.5)	36.3 (14.9)	0.25^*^	28.0 (6.62)	51.7 (8.01)	3.25^**^
Situational impact	18.3 (5.61)	18.8 (6.19)	−0.08	18.6 (5.71)	18.1 (5.83)	0.07
Anticipation	18.1 (6.98)	19.4 (7.00)	−0.18	19.0 (7.08)	17.7 (6.86)	0.19^*^
Modulation	17.2 (7.73)	17.5 (8.10)	−0.05	18.4 (8.00)	15.9 (7.41)	0.33^**^
MIDc total score	53.6 (18.0)	55.7 (19.3)	−0.12	56.0 (18.2)	51.7 (18.2)	0.24^*^
PSQI total score	7.28 (3.83)	7.01 (3.47)	0.07	7.00 (3.30)	7.41 (4.22)	−0.11

** p < 0.01;

* p < 0.05; χ^2^, chi-square; d, Cohen d; MIDc, Mental Impact and Distress: COVID-19; PSQI, Pittsburgh Sleep Quality Index.

### Factor structure of the MIDc and PSQI

The 3-factor CFA model provided an adequate fit to the MIDc (CFI = 0.963, RMSEA = 0.060, and SRMR = 0.045). All of the MIDc items showed substantial loadings on their respective factor (situational impact: λ = 0.58–0.81; anticipation: λ = 0.63–0.90; modulation: λ = 0.71–0.90). The three first-order factors loaded substantially (λ = 0.80–0.95) on the second-order MIDc factor. The 1-factor CFA model provided an adequate fit to the PSQI (CFI = 0.979, RMSEA = 0.069, and SRMR = 0.037) and had substantial factor loadings (λ = 0.45–0.82).

### Comparison across quarantine experience

As shown in Table 2, respondents with quarantine experience were significantly more likely to know confirmed cases and less likely to be the primary income source of the family than those without quarantine experience. Quarantine experience was significantly associated with higher MIDc and PSQI total scores (*d* = 0.23–0.43, *p* < 0.05). For PSQI subscales, quarantine experience was associated with significantly worse subjective sleep quality, longer sleep latency, and greater sleep disturbance (*d* = 0.29–0.32, *p* < 0.05) but not in the other four subscales (*d* = 0.10–0.16, *p* = 0.15–0.38). Quarantine experience was not significantly associated with other study variables (*p* = 0.08–0.73).

**Table 2:** Demographics and psychological profiles of the sample across quarantine experience.

**Variables**	**Without quarantine (*N =* 329)**	**With quarantine (*N =* 109)**	
**Categorical variable**	***N*** **(%)**	***N*** **(%)**	*χ^2^*
Know confirmed case	85 (25.8)	45 (41.3)	9.36^**^
Frontline work	25 (7.6)	11 (10.1)	0.68
Family primary income	150 (45.6)	30 (27.5)	11.0^**^
PSQI diagnosis	199 (60.5)	76 (69.7)	2.99
Continuous variable	Mean (SD)	Mean (SD)	*d*
Age	39.5 (14.1)	36.8 (13.1)	0.19
Situational impact	18.0 (5.70)	19.5 (5.79)	−0.27^*^
Anticipation	17.7 (6.69)	20.7 (7.44)	−0.43^**^
Modulation	16.5 (7.60)	19.5 (8.15)	−0.38^**^
MIDc total score	52.2 (17.6)	59.7 (19.2)	−0.41^**^
Sleep time (hours)	6.32 (1.44)	6.54 (1.30)	0.15
Subjective sleep quality	1.27 (0.71)	1.48 (0.74)	−0.29^**^
Sleep latency	1.15 (0.95)	1.46 (0.98)	−0.32^**^
Sleep duration	1.36 (0.92)	1.21 (0.87)	0.16
Habitual sleep efficiency	0.49 (0.91)	0.61 (0.97)	−0.13
Sleep disturbance	1.27 (0.58)	1.44 (0.62)	−0.29^*^
Sleep medication	0.27 (0.79)	0.35 (0.85)	−0.10
Daytime dysfunction	1.18 (0.88)	1.31 (0.87)	−0.15
PSQI total score	6.99 (3.65)	7.86 (3.99)	−0.23^*^

** p < 0.01;

* p < 0.05; χ^2^, chi-square; d, Cohen d; MIDc, Mental Impact and Distress: COVID-19; PSQI, Pittsburgh Sleep Quality Index.

### SEM results

**Figure 1:**
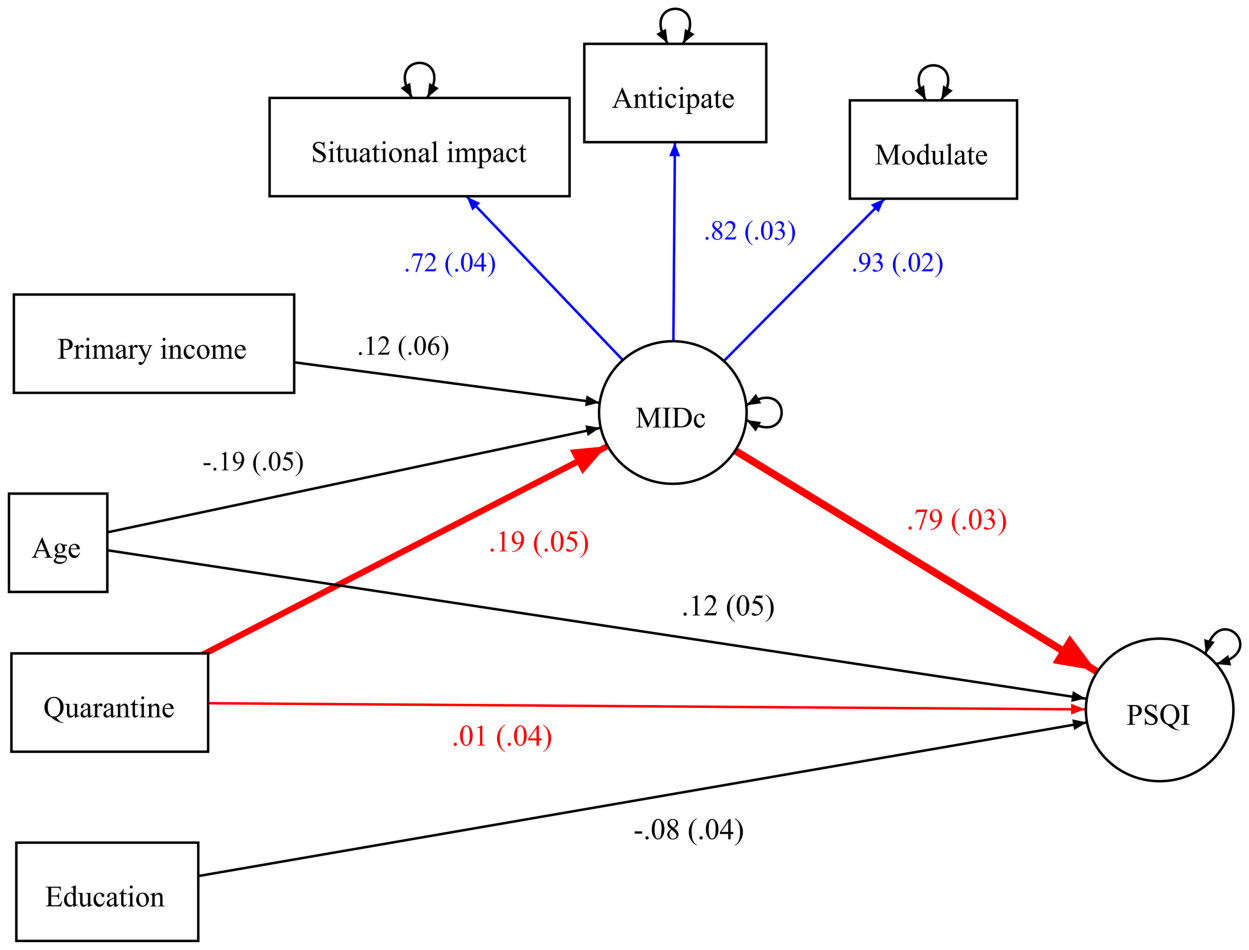
Standardized coefficients of the structural equation model from quarantine experience to PSQI factor *via* MIDc factor. PSQI, Pittsburgh Sleep Quality Index; MIDc, Mental Impact and Distress Scale: COVID-19. Standard errors are presented in parenthesis. The blue, black, and red arrows denoted the factor loadings, covariate effects, and main regression paths, respectively. The indirect pathways from quarantine to PSQI *via* MIDc were highlighted in bold. The seven PSQI observed components and non-significant paths from control variables to MIDc and PSQI were not shown in the figure for the simplicity of presentation.

The SEM on the latent MIDc and PSQI factors provided an adequate fit to the data (CFI = 0.952, RMSEA = 0.050, and SRMR = 0.043). Figure 1 displays the standardized estimates of the SEM from quarantine to the PSQI factor *via* the MIDc factor. The factor loadings for the MIDc factor were highlighted in blue and significant paths from control variables to the study variables were presented in black. The seven PSQI observed components and non-significant paths of control variables were omitted in the figure to simplify the presentation. Being the primary income source of the family and younger were significantly associated with higher MIDc, and being older and having a lower education level were significantly associated with higher PSQI. Gender, knowing confirmed case, and frontline work did not show significant associations with the MIDc factor (*β* = 0.01 to 0.06, SE = 0.05, *p* = 0.19–0.86) and PSQI factor (*β* = −0.06 to −0.01, SE = 0.04, *p* = 0.14–0.86).

Quarantine did not have a significant direct effect (*β* = 0.01, SE = 0.04, *p* = 0.76) on PSQI. There were significant and positive effects (*β* = 0.19–0.79, *p* < 0.01) from quarantine to MIDc and from MIDc to PSQI. The SEM explained 7.8% and 60.7% of the variance of MIDc and PSQI factors, respectively. There was a significant and positive indirect effect (*αβ* = 0.152, 95% CI = 0.071–0.235) from quarantine to PSQI factor *via* MIDc factor, which accounted for 92.1% of the total effect (*αβ* = 0.165, 95% CI = 0.054–0.274) from quarantine to PSQI factor. Subgroup analyses did not reveal significant differences in the indirect effects across gender and age groups.

The SEM on poor sleep quality provided an adequate fit to the data (CFI = 0.965, RMSE*A* = 0.048, and SRMR = 0.031). Quarantine did not have a significant effect (*β* = −0.02, SE = 0.06, *p* = 0.75) on poor sleep quality. The positive effect from quarantine to MIDc factor remained the same (*β* = 0.19, *p* < 0.01) and MIDc factor had a significant and positive effect (*β* = 0.65, SE = 0.05, *p* < 0.01) on poor sleep quality. The direct effect of quarantine on poor sleep quality was not significant (probability = −0.017, 95% CI = −0.122 to 0.090). Quarantine showed a significant and positive indirect effect on poor sleep quality *via* the MIDc factor (probability = 0.107, 95% CI = 0.050 to 0.171).

## Discussion

The present study was the first to investigate the association between quarantine experience and sleep problems among community adults in Hong Kong during the COVID-19 pandemic. Though quarantine experience was not significantly associated with the sleep time and sleep duration in the present sample, quarantine experience was associated with significantly greater interferences in subjective sleep quality, sleep latency, and sleep disturbance and higher levels of MIDc and total PSQI. These results lend support to Hypothesis 1 and are in line with recent findings on elevated levels of social isolation and psychological distress during the COVID-19 pandemic (10, 18–20). More than half (60.5%) of the respondents without quarantine experience reported poor sleep quality while the respondents with quarantine experience showed an even higher prevalence (69.7%). The non-significant difference in the prevalence matches with recent findings where quarantine was significantly associated with psychological distress but not poor sleep quality among individuals in Saudi Arabia (22).

Our model provided empirical support to the inter-relationships among quarantine experience, MIDc, and sleep disturbance during the COVID-19 pandemic. Our results are consistent with recent findings on the detrimental effects of quarantine experience in terms of adverse psychological outcomes, psychological distress, and insomnia symptoms among Chinese people in quarantine (40, 41). The experience of social isolation and loneliness for quarantined individuals could result in higher levels of anticipation and modulation as psychological responses to the COVID-19 pandemic. In the present study, quarantine experience indirectly increased the proportion of poor sleep quality by 10.7% *via* MIDc. The direct effect of quarantine on sleep disturbance became non-significant after controlling for MIDc and MIDc mediated most of the total effect of quarantine on sleep disturbance.

Despite the low number of infected cases in Hong Kong during the 3-month interval, the overall prevalence of sleep problems was relatively high compared to previous local studies (15–17) and studies in other countries (18, 21–26). The Hong Kong government has been criticized for the slow and incomprehensive responses for COVID-19, inconsistent pandemic policies, and acting for political rather than health motives (42). The extent of public trust in the policies implemented by the government could be an important factor for the residents to maintain their mental wellbeing (43, 44). These contextual factors have been associated with poorer mental health (28). Our results contribute to the literature by elucidating an indirect pathway from quarantine to sleep disturbance *via* MIDc during the pandemic. These results support Hypothesis 2 and explicate the mediating role of mental impact and distress associated with COVID-19 in the relationship between quarantine and sleep disturbance.

A recent study (45) established close linkages among symptoms of psychological distress and sleep disturbance *via* network analysis. Recent studies (46, 47) have elaborated on the neurophysiological mechanisms *via* the HPA axis and prefrontal cortex to explain the potential implications of quarantine experience on psychological health. The quarantine experience could be a stressful event and the perception of threat and fear likely results in amygdala activation (48), which has been linked to perceived stress in the context of COVID-19 pandemic (49). Several studies (50–52) demonstrated elevated symptoms of post-traumatic stress disorder (PTSD) among Chinese samples undergoing quarantine during the pandemic. A recent systematic review (53) suggests shared neuromodulatory pathways between PTSD and sleep disturbance and PTSD symptoms could disrupt individuals' sleep patterns during both rapid eye movement (REM) and non-REM sleep. Moreover, clusters of electroencephalography (EEG) data was found to differentiate psychological distress and sleep disturbance in 59 Australian adolescents (54). Further EEG-based studies should examine the neurophysiological factors associated with MIDc and sleep disturbance.

From a practical perspective, our findings facilitate the formulation of interventions toward recovery or adjustment to a new normal following the pandemic. Governments have the mandate to better prepare and learn from the experience of previous pandemics to build a better capacity to deal with upcoming crises in terms of normalization and adaptation (55). Our results indicate that to achieve effective improvements in sleep quality, the levels of pandemic-specific distress need to be assessed and addressed at the same time. Social comparison on social networking sites has been found to ameliorate the increase in loneliness and psychological distress during the COVID-19 quarantine (56). It is essential to use social support as a coping mechanism against social isolation and loneliness (57). Web-based or app-based interventions could make use of modern technological access to help quarantined persons remain socially active to mitigate their feelings of social isolation.

### Study limitations

The present study has several limitations. First, the online survey recruited participants *via* mass emails and websites under convenience sampling. It was not possible to calculate the survey response rate as we could not know the number of potential participants who read the mass email and websites. There could be self-selection bias in the recruitment such that community adults with greater sleep disturbance were more inclined to join the survey. The non-random sampling design limits the generalizability of the findings. Further studies are required to verify the robustness of the results across different age and cultural groups. Second, the cross-sectional study design prevents inferences of the causal direction in the relationships and sleep disturbance could have reciprocal effects on COVID-19 mental impact and distress. Longitudinal studies are needed to clarify directional relationships among the study variables over a more extended period in the context of the pandemic.

Third, our results could be subject to omitted variables and confounding biases. The present study did not assess details of quarantine experience such as the isolation period, time after isolation, and reason of quarantine. These factors and other restriction policies such as social distancing and vaccine mandate might influence the present results. Further qualitative studies are needed to understand the impact of quarantine experience on the individuals. Fourth, sleep quality has been found to be associated with physical activity (58) and sedentary behavior (59) during COVID-19 quarantine. Future studies are needed to elucidate the mediating role of sleep disturbance in the relationships between behavioral indicators and mental health outcomes during the quarantine. Such research could provide useful findings in formulating targeted interventions on lifestyle modifications to mitigate the adverse effects of social isolation on better psychological wellbeing.

## Conclusion

The present study contributes to a more nuanced understanding of the effects of quarantine experience on sleep disturbance of Hong Kong adults during the COVID-19 pandemic. The results support indirect effects from quarantine experience to greater sleep disturbance *via* mental impact and distress associated with COVID-19. It is essential for researchers, practitioners, and policymakers to conduct further evaluations on how to better support the quarantined individuals and to mitigate the potential adverse effects of quarantine experience on mental well-being and sleep quality from the public health perspective.

## Data availability statement

The original dataset presented in the study are included in the article/Supplementary material, further inquiries can be directed to the corresponding author.

## Ethics statement

The studies involving human participants were reviewed and approved by Human Research Ethics Committee of the University of Hong Kong (Reference number = EA210291). The patients/participants provided their written informed consent to participate in this study.

## Author contributions

Conceptualization and methodology: TF, KC, and RH. Formal analysis and writing–original draft: TF. Investigation and project administration: TF and KC. Writing–review and editing: KC and RH. Resources and supervision: RH. All authors read and approved the final manuscript.
